# Effect of stellate ganglion block on the prevention of posttraumatic stress disorder in patients undergoing emergency ocular trauma surgery: protocol for a randomized, double-blind, placebo-controlled trial

**DOI:** 10.3389/fmed.2025.1663506

**Published:** 2025-10-02

**Authors:** Qi Gao, Bin Liu, Zhengjie Chen, Li Li, Ning Li, Gerong Zhang, Shuo Di, Yangzi Zhu, Lei Zhu, Youjia Yu

**Affiliations:** ^1^Department of Anesthesiology, The First Central Hospital of Baoding, Baoding, Hebei, China; ^2^Department of Anesthesiology, The Hospital of 82ND Group Army PLA, Baoding, Hebei, China; ^3^Department of Anesthesiology, Sir Run Run Shaw Hospital, School of Medicine, Zhejiang University, Hangzhou, Zhejiang, China; ^4^Department of Anesthesiology, Xuzhou Central Hospital, Xuzhou, Jiangsu, China; ^5^Jiangsu Province Key Laboratory of Anesthesiology, Xuzhou Medical University, Xuzhou, Jiangsu, China; ^6^Key Laboratory of Molecular Pathology and Early Diagnosis of Tumor in Hebei Province, Baoding, China; ^7^Department of Anesthesiology, Suzhou Xiangcheng People’s Hospital, Suzhou, Jiangsu, China

**Keywords:** stellate ganglion block, posttraumatic stress disorder, emergency, ocular trauma, protocol

## Abstract

**Background:**

Post-traumatic stress disorder (PTSD) is a prevalent and debilitating mental health condition that often develops after exposure to traumatic events. Stellate ganglion block (SGB) has been shown to alleviate PTSD symptoms, suggesting its potential as a preventive intervention, particularly in patients undergoing emergency ocular trauma surgery. However, the efficacy of SGB in preventing the onset of PTSD has not been clearly established.

**Methods:**

This dual-center, randomized, double-blind, placebo-controlled trial will enroll 300 adult patients undergoing emergency ocular trauma surgery. Participants will be randomly assigned, in a 1:1 ratio and stratified by age (<65 or ≥65 years), to either the SGB group or the placebo group (*n* = 150 per group). Each participant will receive either an active right stellate ganglion block or a sham procedure administered 15 min prior to the induction of anesthesia. The primary outcome is the difference in the incidence of PTSD at 1 month after surgery. Secondary outcomes include the severity of PTSD, delayed-onset PTSD, the four core symptom clusters (intrusive re-experiencing, avoidance, negative alterations in cognition or mood, and hyperarousal and reactivity), the severity of dissociative symptoms, Beck Anxiety Inventory (BAI) scores at 24, 48, and 72 h postoperatively; Visual Analog Scale (VAS) sleep scores at 24, 48, and 72 h postoperatively; Numerical Rating Scale (NRS) pain scores at 24, 48, and 72 h postoperatively; Heart Rate Variability (HRV) measured intraoperatively and at 24 and 48 h postoperatively; recovery time; extubation time; Richmond Agitation-Sedation Scale (RASS) scores; and length of hospital stay. Safety outcomes will include neck pain, dizziness, tinnitus, respiratory depression, anaphylaxis, sinus bradycardia (defined as heart rate <50 beats/min), hematoma formation, infection, severe arrhythmia, pneumothorax, and complications related to general or spinal anesthesia. All data will be analyzed using a modified intention-to-treat (mITT) approach.

**Discussion:**

This study aims to evaluate the efficacy and safety of SGB for the prevention of PTSD in patients undergoing emergency ocular trauma surgery.

**Clinical trial registration:**

ChiCTR2500102717, www.chictr.org.cn/showproj.html?proj=270046.

## Introduction

Post-traumatic stress disorder (PTSD) is a mental health condition that develops in individuals following exposure to threatening, severe, or catastrophic events. Clinically, it is characterized by symptoms such as hypervigilance, delayed reactions, and intentional avoidance of trauma-related stimuli (1, 2). Epidemiological studies report a lifetime prevalence of PTSD ranging from 13.0 to 20.4% in women and from 6.2 to 8.2% in men (3). The incidence of PTSD following trauma-related hospitalization has been reported to be as high as 23% (4). Once diagnosed, PTSD not only affects a patient’s psychological well-being but also has detrimental effects on physical health, social relationships, and occupational functioning. It significantly impairs quality of life and increases the risk of comorbid mental disorders such as depression and suicide (5, 6).

Ocular trauma refers to mechanical, physical, or chemical injuries to the eye and its associated structures, resulting in structural and/or functional impairment (7). In China, the incidence of ocular trauma is relatively high, with an estimated 5 to 12 million new cases reported annually (8). Patients with ocular trauma often experience varying degrees of visual impairment, and visual deprivation may disrupt spatial orientation and intensify feelings of fear (9). Additionally, trauma-induced pain can reinforce traumatic memories through mechanisms of central sensitization, while visible facial injuries may contribute to emotional distress, increasing the risk of anxiety, depression, and PTSD in these patients (10, 11). Studies have shown that up to 33% of individuals with ocular trauma may develop PTSD (9, 11). Therefore, early and timely intervention is crucial for reducing the risk of PTSD in this population.

Stellate ganglion block (SGB) involves the injection of a local anesthetic into the stellate ganglion at the base of the neck to temporarily block sympathetic nerve activity (12). It is thought to exert its therapeutic effects by modulating the sympathetic nervous system through cervical sympathetic blockade (13). While most previous studies (14–18) have investigated SGB as a treatment for established PTSD, the disorder is often chronic and difficult to manage once it develops, even with available therapies (3, 14). Preventive intervention may therefore offer greater benefit, particularly in high-risk populations such as patients with ocular trauma. The acute perioperative period represents a critical window for fear memory consolidation, and timely modulation of sympathetic activity may disrupt this process and reduce the likelihood of PTSD onset. Moreover, ultrasound-guided SGB is a rapid, safe, minimally invasive, and cost-effective procedure (19), supporting its potential as a practical preventive strategy in this setting. Our group has accumulated relevant experience in this field. We previously investigated preventive strategies for PTSD in patients undergoing emergency trauma surgery (20–22), which provided valuable insights into study design and the perioperative application of SGB. In addition, our preclinical work demonstrated that SGB could attenuate the consolidation of conditioned fear memory by modulating the locus coeruleus–basolateral amygdala circuit, thereby offering mechanistic support for its use in early trauma settings (20). Collectively, these prior studies provide both methodological and biological foundations for the present trial, reinforcing its reliability and feasibility.

This study aims to assess the efficacy and safety of preoperative SGB in reducing the incidence of PTSD in patients undergoing emergency ocular trauma surgery.

## Methods

This protocol follows to the Standard Protocol Items: Recommendations for Interventional Trials (SPIRIT) guidelines, which is detailed in Supplementary file 1.

### Study design and patients

**Figure 1:**
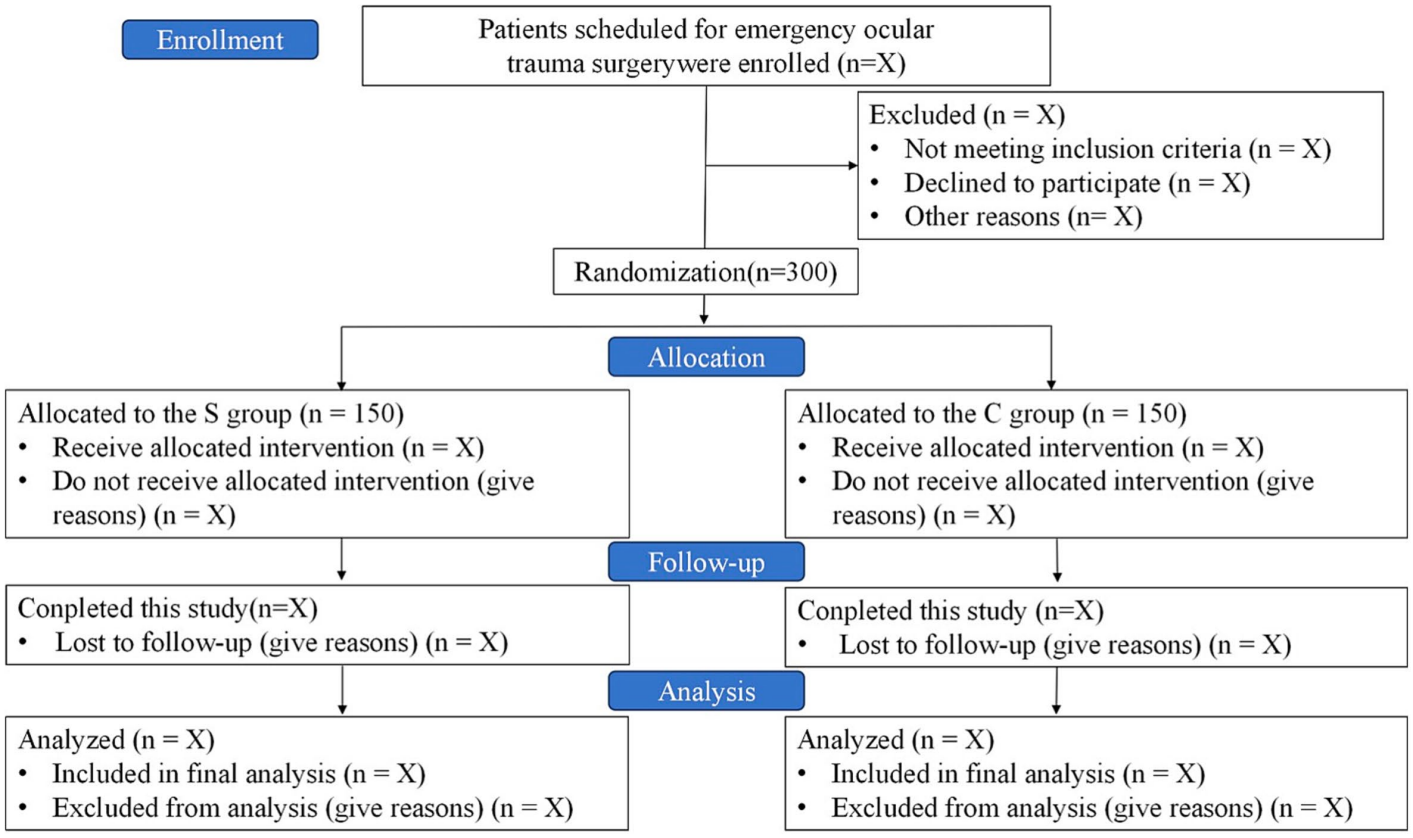
Study flow diagram. SGB, stellate ganglion block.

This is a dual-center, prospective, randomized, patient- and assessor-blinded, parallel-group, controlled clinical trial. A total of 300 patients will be recruited from the First Central Hospital of Baoding and the 82nd Group Army Hospital of the People’s Liberation Army (PLA). Recruitment is scheduled to take place from May 22, 2025, to November 22, 2025. Follow-up period is from November 23, 2025, to May 22, 2026. A detailed study flow diagram is shown in Figure 1.

### Inclusion criteria

Patients who meet the following criteria will be included:

- Age 18 or older.
- Acute unilateral or bilateral ocular trauma and scheduled for surgery under general anesthesia.
- American Society of Anesthesiologists (ASA) physical status I-III E.
- Provided informed consent, with approval from both patients and their families.

### Exclusion criteria

The exclusion criteria include:

- A known allergy to ropivacaine.
- Infection of the skin on the neck.
- Combine severe neurological or mental disorders unable to undergo assessmtent.
- Craniocerebral or spinal cord injury.
- Shock decompensation.
- Associated with multiple and complex injuries and requiring concurrent implementation of multiple surgical procedures.
- Severe atrioventricular block or bradyarrhythmia with a baseline heart rate lower than 50 beats/min.
- Severe visual, hearing, or speech impairments that prevent the completion of assessment.

### Primary outcome

The primary outcome is the difference in the incidence of PTSD between the two groups at 1 month postoperatively, assessed using the Clinician-Administered PTSD Scale for DSM-5 (CAPS-5). The CAPS-5 is a structured diagnostic interview widely regarded as the gold standard for PTSD assessment. Neuropsychological evaluations will be conducted by professionally trained anesthesiologists who are blinded to group allocation and will perform assessments in a calm and controlled environment. Data analysts will also remain blinded to treatment assignments. The CAPS-5 provides both a diagnostic determination (presence or absence of PTSD) and a continuous measure of PTSD severity, including the four core symptom clusters: intrusions, avoidance, negative alterations in cognition and mood, and arousal/reactivity. The assessment can be administered by appropriately trained clinicians or paraprofessionals.

### Secondary outcomes

Secondary outcomes include the severity of PTSD, delayed-onset PTSD, the four core symptom clusters (intrusive re-experiencing, avoidance, negative alterations in cognition or mood, and hyperarousal/reactivity), and the severity of dissociative symptoms. Other secondary outcomes include Beck Anxiety Inventory (BAI) scores (range 0–84, higher scores indicating greater anxiety) assessed at 24, 48, and 72 h postoperatively; Visual Analog Scale (VAS) sleep scores (range 0–10, with 0 representing best and 10 worst sleep quality) at the same time points; and Numerical Rating Scale (NRS) pain scores (range 0–10, higher scores indicating more severe pain) also at 24, 48, and 72 h. Heart Rate Variability (HRV) will be assessed intraoperatively and again at 24 and 48 h postoperatively. Recovery time is defined as the interval from cessation of general anesthesia maintenance medication to recovery of consciousness, while extubation time is defined as the interval from cessation of anesthesia maintenance medication to extubation. The Richmond Agitation-Sedation Scale (RASS) will be used to evaluate patients’ level of consciousness, sedation, agitation, and their severity. Finally, the length of hospital stay will also be recorded as a secondary outcome.

### Safety outcomes

Safety outcomes will include the occurrence of both common and serious intraoperative or postoperative adverse events. Common adverse events consist of neck pain, dizziness, tinnitus, and mild allergic reactions, which will be evaluated by combining patient complaints with clinical observation. Serious adverse events include respiratory depression, defined as a sustained SpO_2_ of less than 90% or the need for assisted ventilation; anaphylaxis, defined as the development of anaphylactic shock; sinus bradycardia, defined as a heart rate of less than 50 beats per minute; hematoma formation; infection; severe arrhythmias; pneumothorax; and complications related to general or spinal anesthesia.

### Randomization and blinding

An independent research staff member will generate the randomization sequence using the Sealed Envelope online tool,^1^ with a 1:1 allocation ratio, permuted blocks of 2 and 4, and stratification by age (18–64 and ≥65 years). The allocation results will be stored in sequentially numbered, sealed opaque envelopes. Patients will be randomly assigned to either the SGB group or the control group (*n* = 150 per arm). The anesthesiologists responsible for administering the intervention will be aware of group allocation due to the nature of the procedure but will not be involved in patient recruitment, data collection, or outcome assessment. Patients, surgeons, postoperative care providers, outcome assessors, and the data analyst will remain blinded to group assignments throughout the study. Successful stellate ganglion block in the intervention group will be confirmed by the occurrence of Horner’s syndrome (ptosis, miosis, enophthalmos, conjunctival hyperemia, and facial anhidrosis). Patients will not be informed whether these clinical signs occur, thereby maintaining the integrity of blinding. The schedule for patient enrollment, study interventions, and outcome assessments will adhere to the SPIRIT statement (Table 1).

**Table 1:** SPIRIT schedule of patient enrolment, study interventions and outcome assessment.

Time point	Study period									
	Enrolment	Allocation	Post-allocation					Close-out	Follow-up	
	Emergency room	15 min Pre-an	Intraoperative	Post-op	24 h Post-op	48 h Post-op	72 h post-op	Discharged	1 month	6 months
Patient enrolment										
Eligibility criteria	**×**									
Written informed consent	**×**									
Demographic data	**×**									
Baseline characteristics	**×**									
Randomization/allocation	**×**									
Study interventions										
SGB		**×**								
Sham SGB		**×**								
Outcome assessment										
CAPS-5 score									**×**	**×**
BAI score					**×**	**×**	**×**			
VAS Sleep score					**×**	**×**	**×**			
NRS Pain score					**×**	**×**	**×**			
HRV			**×**		**×**	**×**				
Recovery time				**×**						
Extubation time				**×**						
RASS score				**×**						
Length of hospital stay								**×**		
Safety Outcomes										
Neck pain			**×**		**×**	**×**	**×**			
Dizziness			**×**		**×**	**×**	**×**			
Tinnitus			**×**		**×**	**×**	**×**			
Dyspnea			**×**		**×**	**×**	**×**			
Anaphylaxis			**×**		**×**	**×**	**×**			
Sinus bradycardia			**×**		**×**	**×**	**×**			
Hematoma formation			**×**		**×**	**×**	**×**			
Infection			**×**		**×**	**×**	**×**			
Severe arrhythmia			**×**		**×**	**×**	**×**			
Pneumothorax			**×**		**×**	**×**	**×**			
General spinal anesthesia			**×**		**×**	**×**	**×**			

SGB, Stellate Ganglion Block; CAPS-5, the Clinician-Administered PTSD Scale for Diagnostic and Statistical Manual of Mental Disorders (Fifth Edition); BAI, Beck Anxiety Inventory; VAS, Visual Analogue Scale; NRS, Numerical Rating Scale; HRV, Heart Rate Variablity; RASS, Richmond Agitation Sedation Scale; Sinus bradycardia, defined as HR<50 beats/min; Pre-an, Preanesethesia; Post-op, Postoperative; SPIRIT, Standard Protocol Items: Recommendations for Interventional Trials.

### Study interventions

Patients will receive either a right SGB or a sham procedure 15 min prior to induction of anesthesia. In the SGB group, 5 mL of 0.5% ropivacaine will be injected around and into the stellate ganglion at the level of the C6 anterior tubercle under ultrasound guidance (23). In the control group, 2 mL of preservative-free normal saline will be injected into the deep musculature anterolateral to the anterior tubercle of C6, deliberately avoiding the stellate ganglion. The injection of 2 mL saline into a region other than the stellate ganglion is mainly for evaluating the placebo effects of the study procedures. Apart from the injectate and its site of deposition, all other procedures, including patient preparation, positioning, ultrasound guidance, and needle insertion technique, will be identical in both groups.

### Intervention implementation process

Patients will be placed in the supine position with the head slightly rotated to the left. A thin pillow will be positioned under the shoulders to slightly extend the neck and optimize exposure. At the level of the C6 vertebra, between the trachea and the carotid artery, a high-frequency linear ultrasound probe will be used to gently displace the carotid artery laterally, allowing visualization of the transverse process of C6 with its prominent anterior and posterior tubercles, as well as the longus colli muscle located superficial to the transverse process and covered by the prevertebral fascia. Using an in-plane technique, the needle will be advanced under real-time ultrasound guidance. It will traverse the sternocleidomastoid and anterior scalene muscles, then pass into the space between the anterior tubercle of the C6 transverse process and the internal jugular vein, reaching the anterior surface of the longus colli muscle beneath the prevertebral fascia. If no blood is aspirated, the study solution will be injected. All procedures will be performed under sterile conditions by an experienced anesthesiologist, using real-time ultrasound guidance. The presence of ipsilateral Horner’s syndrome is considered an indicator of successful blockade.

### Anesthesia procedures and perioperative management

All patients enrolled in this trial will undergo general anesthesia. Intraoperative monitoring will include non-invasive blood pressure (NIBP), electrocardiography (ECG), pulse oximetry (SpO_2_), and bispectral index (BIS). Anesthesia will be induced with intravenous propofol (1.5–2.5 mg/kg), combined with sufentanil (0.2–0.4 μg/kg) for analgesia. Rocuronium (0.6–0.9 mg/kg) will then be administered to facilitate muscle relaxation. Following induction, endotracheal intubation will be performed, and tube placement will be confirmed. Mechanical ventilation will be initiated to maintain end-tidal carbon dioxide between 35 and 45 mmHg.

Anesthesia maintenance will consist of continuous infusions of propofol (2–4 mg/kg/h) and remifentanil (0.1–0.2 μg/kg/min), along with 1% sevoflurane. BIS values will be maintained between 40 and 60. Intraoperative hypotension (mean arterial pressure <65 mmHg or a ≥20% decrease from baseline) and bradycardia (heart rate <50 beats/min) will be treated as needed.

All patients will receive 8 mg of ondansetron to prevent postoperative nausea and vomiting. In the postoperative ward, if the VAS pain score exceeds 4, 50 mg of intravenous flurbiprofen axetil will be administered for analgesia.

### Data collection and monitoring

Patient data will be thoroughly reviewed by the research team using individual electronic medical records. The collected baseline variables will include age (years), sex, body mass index (BMI, kg/m^2^), education level, preoperative medications, comorbidities, American Society of Anesthesiologists (ASA) physical status classification, smoking status, and the time from injury to anesthesia induction, among others. Post-discharge information will be obtained via direct interviews or telephone follow-ups and recorded in the follow-up management system. In addition, major life events such as serious illnesses, accidents, or the death of close family members will also be documented.

All data will be recorded on case report forms (CRFs) and entered into a secure electronic database under the supervision of the principal investigator. An independent Data Monitoring Committee (DMC) will oversee the data collection process throughout the study. Upon completion of data entry, the database will be locked and anonymized datasets will be submitted to an independent statistician for final analysis according to the predefined statistical analysis plan.

Any serious adverse events (SAEs), whether related or unrelated to the study intervention, must be reported immediately to the principal investigator. Appropriate medical measures will be initiated by the perioperative care team to ensure patient safety. In addition, all SAEs will be reported to the DMC within 24 h for evaluation, including assessment of whether protocol modifications or study termination is required.

### Sample size calculation

According to the literature, the incidence of PTSD at 1 month after trauma surgery is approximately 23.2% (21). Previous epidemiological studies have reported rates up to 30–33% in patients with ocular trauma, substantially higher than in the general trauma population (9, 11). Based on these data and our small pilot observation, we set the expected PTSD incidence in the control group at 26%. We therefore adopted 26% as a conservative estimate to guide the sample size calculation. This conservative approach ensures that statistical power will not be underestimated if the true incidence in our study population is somewhat lower than that reported in epidemiological studies, while still remaining consistent with the published range. We hypothesize that SGB will reduce the risk of postoperative PTSD by 50% in patients undergoing emergency ocular trauma procedures. Using a two-sided test with a significance level of *α* = 0.05 and 80% power, a minimum of 142 participants per group is required. Accounting for an anticipated 10% dropout rate, the final target sample size is 300 patients, with 150 allocated to each group.

### Statistical analysis

The normality of continuous variables will be evaluated using the Shapiro–Wilk test. Data following a normal distribution will be reported as mean (standard deviation, SD), while non-normally distributed data will be expressed as median (interquartile range, IQR). Group comparisons for normally distributed continuous variables will be conducted using independent t-tests or repeated measures ANOVA, as appropriate. For non-normally distributed variables, analyses will be performed using the Mann–Whitney U test or generalized estimating equations (GEE) to account for repeated measures and potential correlations. Categorical data will be presented as counts (percentages) and analyzed using the chi-square test or Fisher’s exact test, as appropriate, based on expected frequency distributions.

The 1-month postoperative prevalence of PTSD, defined as the primary endpoint, will be compared between groups using the chi-square (*χ*^2^) test. Group differences will be reported as odds ratios (ORs) with 95% confidence intervals (CIs). To control for potential confounders, logistic regression models will be used to obtain adjusted ORs. Subgroup analyses will also be conducted using logistic regression to examine treatment-by-covariate interactions.

Analyses of the primary outcome will primarily follow a modified intention-to-treat (mITT) approach, including all randomized participants with available data, regardless of protocol adherence. A per-protocol (PP) analysis will also be performed, excluding patients with major protocol deviations or who withdrew consent, to assess the treatment effect among fully adherent participants. No multiple testing correction is planned for secondary outcomes; these results will be interpreted as exploratory.

To evaluate CAPS-5 score changes over time (at 1- and 6-month follow-ups), repeated measures analysis will be performed using a covariate-adjusted linear mixed model (LMM). Fixed effects will include group (SGB vs. control), time (1 month vs. 6 months), and the group-by-time interaction. Potential confounding variables—such as sex, age, Injury Severity Score (ISS), laterality of ocular trauma (unilateral or bilateral), time from injury to surgery, and baseline VAS pain scores—will be entered as covariates. If significant time-by-covariate interactions are detected, they will be incorporated into the model. Results will be presented as least squares means (LSMs) with 95% CIs.

Sensitivity analyses will be conducted to evaluate the robustness of the findings. These will include complete case analysis and multiple imputation for handling missing data, additional covariate-adjusted regression models, and subgroup analyses to explore the consistency of treatment effects under different modeling assumptions.

As exploratory endpoints, Spearman’s or Pearson’s correlation analyses will be used to assess the relationships between preoperative VAS pain scores or Injury Severity Scores (ISS) and postoperative CAPS-5 scores.

All hypothesis testing will be two-sided, with a significance level of *p* < 0.05. For subgroup interaction terms, a threshold of *p* < 0.10 will be used to indicate potential effect modification. All statistical analyses will be performed using SPSS software (version 25.0; IBM Corp., Armonk, NY, United States). No interim analysis will be conducted.

### Patient and public involvement

Patients and members of the public will not be involved in the design, recruitment, implementation, or reporting of this study. Study findings will be communicated to participants via email upon completion.

### Principles and methods of unblinding or breaking the blind

#### Unblinding timeline

All participants will be unblinded upon the study’s completion, specifically after the final 6-month follow-up for all subjects.

#### Unblinding method

The unblinding process will be managed by an independent Data Monitoring Committee (DMC), which will oversee the procedure and securely store all randomization data until the predetermined unblinding period.

#### Emergency unblinding

In the event of a serious adverse event (SAE) or any other emergency during the trial, immediate unblinding will be conducted to facilitate appropriate medical intervention. The principal investigator will formally request emergency unblinding from the DMC, ensuring that both the rationale and the process are thoroughly documented.

## Discussion

The randomized, double-blind, placebo-controlled trial, involving 300 adults at two centers, was designed to evaluate SGB for the prevention of PTSD after emergency ocular trauma. The primary objective is to evaluate the effect of SGB prevention in patients with ocular trauma by the incidence of PTSD 1 month after surgery. Secondary objectives include the severity of PTSD, delayed-onset PTSD, four symptom clusters, the severity of dissociative symptoms, as well as BAI score, VAS sleep score, NRS pain score, HRV, recovery time, extubation time, and RASS score. This study will be conducted in accordance with the Consolidated Standards of Reporting Trials (CONSORT) guidelines (24). To enhance the credibility of the primary outcome, baseline variables were adjusted, and sensitivity analyses were conducted to evaluate the stability of the findings. These methodological strategies were implemented to ensure the accuracy, consistency, and reliability of the results.

The stellate ganglion, also known as the cervicothoracic sympathetic ganglion, is part of the cervical sympathetic chain and is formed by the fusion of the inferior cervical and first thoracic ganglia in approximately 80% of individuals (25, 26). Its anatomical location is relatively constant, typically situated anterior to the C7 transverse process and superior to the neck of the first rib (25, 26). Stellate ganglion block (SGB) exerts its therapeutic effect by injecting local anesthetics near the stellate ganglion to inhibit peripheral sympathetic nerve activity. Several studies have demonstrated the effectiveness and safety of SGB in alleviating PTSD-related symptoms (14–18). In a cohort of 166 military personnel reported by Dr. Mulvaney et al., approximately 70% of those who received SGB at the C6 level experienced a significant reduction in PTSD Checklist (PCL) scores, with benefits persisting for 3 to 6 months post-procedure (27). Furthermore, a multicenter randomized clinical trial involving military patients with PTSD found that repeated SGB procedures, administered biweekly, led to improvements in symptoms such as depression, anxiety, pain, physical function, and overall psychological well-being (23).

In this study, we will perform a right stellate ganglion block under ultrasound guidance. Ultrasound-guided SGB is a safe and effective method (19). The right side was chosen on the basis of functional hemispheric lateralization, as previous studies have demonstrated right-sided dominance of the sympathetic nervous system and neural circuits related to fear and anxiety (23, 28, 29). Unilateral blockade is sufficient to modulate sympathetic activity while minimizing the risk of complications associated with bilateral procedures, such as recurrent laryngeal nerve palsy or phrenic nerve paralysis.

This randomized controlled trial investigates the potential of SGB to prevent PTSD in patients undergoing emergency ocular trauma surgery. Mechanistically, PTSD is characterized by chronic overactivation of the sympathetic nervous system, which promotes excessive consolidation of fear memories, manifesting clinically as recurrent flashbacks and hypervigilance (30, 31). This hyperadrenergic state is thought to be driven by elevated levels of nerve growth factor (NGF), which facilitates sympathetic sprouting and increases systemic norepinephrine (NE). SGB may counteract this process by downregulating NGF and/or NE, thereby disrupting the pathological sympathetic feedback loop and mitigating the development of PTSD-related symptoms (32). Our previous preclinical study further supports this hypothesis, demonstrating that SGB attenuated conditioned fear memory formation in mice by inhibiting noradrenergic projections from the locus coeruleus to the basolateral amygdala (20).

Visual deprivation resulting from ocular trauma may enhance emotional sensitivity and facilitate the encoding of fear-related memories, possibly through heightened activation of the amygdala, a key brain region involved in emotional processing and memory consolidation (33, 34). SGB may exert its preventive effect on PTSD by inhibiting sympathetic outflow at the stellate ganglion level, thereby attenuating excessive amygdala activation and potentially disrupting the early consolidation of fear memories following trauma (20, 31).

This study has several limitations. First, the follow-up duration is limited to 6 months, which may not be sufficient to determine the long-term sustainability of SGB’s preventive effect on PTSD. Future studies could evaluate long-term efficacy by extending follow-up to 12 months or beyond. Second, the intervention involves a single SGB procedure. Although feasible in emergency settings, this design precludes evaluation of cumulative or repeated-block protocols that may produce more robust or durable outcomes. The dose–response relationship thus remains unclear. Third, while heart rate variability (HRV) is included as an objective physiological measure, other mechanistic markers such as plasma catecholamine levels or functional neuroimaging (for example, amygdala activity on fMRI) are not assessed. Finally, the study population is restricted to adults with ocular trauma treated at two centers in China, which may limit the generalizability of the findings to other trauma types, healthcare systems, or cultural contexts.

The primary purpose of this study is to evaluate whether SGB, administered during the acute perioperative period of ocular trauma surgery, can prevent the onset of PTSD. Unlike previous research that has mainly focused on SGB as a treatment for chronic PTSD, our trial targets its potential as a preventive intervention during the critical early window of fear memory consolidation. Patients with ocular trauma represent a uniquely high-risk group, as sudden vision threat, appearance changes, and functional loss markedly increase vulnerability to PTSD. Current strategies for prevention rely largely on psychosocial support delivered after discharge, and effective biological interventions are lacking. By targeting sympathetic hyperactivity, SGB may disrupt pathological memory formation and offer a rapid, low-cost, and feasible approach that can be seamlessly integrated into routine trauma surgery workflows. If successful, this study could not only establish SGB as a preventive strategy in high-risk trauma populations but also provide a new framework for incorporating early biological interventions into acute surgical care.

## Footnotes

^1^ https://www.sealedenvelope.com/simple-randomiser/v1/lists
